# Deciphering the immunological landscape of HR + metastatic breast cancer: insights from single-cell transcriptomics

**DOI:** 10.1007/s13577-026-01384-2

**Published:** 2026-05-08

**Authors:** Lifang He, Qianqian Zhao, Zexiao Chen, Lijuan He, Zhaochang Qi, Jundong Wu, Kexiang Zhou, Yukun Cui

**Affiliations:** 1 Breast Center, Cancer Hospital of Shantou University Medical College, 7 Raoping Road, Shantou, 515041 Guangdong China; 2 Laboratory for Breast Cancer Diagnosis and Treatment, Shantou University Medical College, Cancer Hospital of Shantou University Medical College, 7 Raoping Road, Shantou, 515041 Guangdong China; 3 School of Computer Science and Technology, Harbin University of Science and Technology, Harbin, Heilongjiang China; 4 Outpatient Nursing, Hangzhou West Lake Zhijiang Ophthalmology Hospital, Hangzhou, Zhejiang China; 5 The Third Affiliated Hospital of Chongqing Medical University, No. 1, Shuanghu Branch Road, Huixing Street, Yubei District, Chongqing, 401120 China

**Keywords:** Breast cancer, Ovarian metastasis, Single-cell sequencing, Tumor microenvironment, Hormone receptor-positive

## Abstract

Breast cancer remains the leading cause of cancer-related mortality among women worldwide, primarily due to metastatic complications. The immune components of the tumor microenvironment (TME) significantly influence metastatic progression. The objective of this study was to uniquely characterize the TME of hormone receptor-positive (HR +) breast cancer with a focus on ovarian metastasis using single-cell RNA sequencing. We delineated the cellular architecture of breast cancer tissues. A total of 9 cell types in 18 clusters were identified, including T cell, B cell and plasma, macrophage, neutrophil, fibroblast, macrophage, endothelial, basal, luminal, SMC (smooth muscle cells) and proliferation cells. Furthermore, macrophages were divided into tumor-associated macrophages and monocyte macrophages, with detailed marker gene analysis. Key statistical findings include the identification of five critical genes (GPR183, BHLHE41, CD83, SLC25A37, and SELL) associated with macrophage functionality. In vivo validation using immunohistochemistry on clinical samples from 10 breast cancer patients with ovarian metastases confirmed that GPR183, BHLHE41, and CD83 were highly expressed in tumor tissues, while SLC25A37 and SELL were highly expressed in adjacent normal tissues. Furthermore, survival analysis correlated the expression levels of these genes with patient outcomes, thus presenting potential prognostic biomarkers. Our study contributes to a deeper insight of the tumor microenvironment in HR + metastatic breast cancer, and offers potential targets for developing novel therapeutic interventions aimed at mitigating metastatic progression and improving clinical outcomes for HR + breast cancer patients.

**Supplementary Information:**

The online version contains supplementary material available at 10.1007/s13577-026-01384-2.

## Introduction

Breast cancer remains the most common malignant tumor worldwide, and systemic metastasis is the leading cause of death in these patients [1]. Recent data indicate that breast cancer accounts for 24.5% of all cancer cases and 15.5% of cancer deaths among women globally, underscoring the critical need for effective diagnostic and therapeutic strategies [2]. Ovarian metastasis occurs in approximately 3–30% of cases, often bilaterally, leading to a 5-year survival rate of less than 10% [3]. Hormone receptor-positive (HR +) breast cancers, which express estrogen and/or progesterone receptors, represent 60–70% of all breast cancer cases. This subtype exploits hormonal pathways for primary tumor growth, as well as for establishing and maintaining metastatic sites, including ovarian metastases. This interaction with the ovarian hormonal milieu may promote tumor cell survival and proliferation at the metastatic site, which collectively contribute to the poor prognosis for these patients [4]. Understanding the specific interactions between HR + breast cancer cells and the ovarian environment is crucial for developing immunotherapies and targeted therapies that could disrupt these interactions, thereby improving patient outcomes [5].

The tumor microenvironment (TME) plays a pivotal role in the progression of breast cancer, particularly in the context of metastasis. It comprises various cell types, including immune cells, fibroblasts, and endothelial cells, surrounded by an array of cytokines that can either inhibit or promote tumor growth. This complex network, known as the tumor immune microenvironment, significantly influences tumor behavior and patient outcomes [6]. Macrophages, a key component of the TME, are particularly noteworthy due to their dual roles in cancer progression. Tumor-associated macrophages (TAMs) have been recognized as potential prognostic factors in breast cancer. Their influence on the overall metabolic status of the TME highlights their importance across many types of malignancies, including breast cancer [7]. Clinical studies have demonstrated that immune checkpoint inhibitors, such as a PD-1 inhibitor, significantly benefit patients with triple-negative breast cancer. However, most patients with HR +  breast cancers respond poorly to treatment with a PD-1 inhibitor, highlighting the importance of understanding the TME in HR + breast cancer with ovarian metastasis.

Advances in single-cell DNA sequencing and single-cell RNA sequencing (scRNA-seq) have provided new methods for assessing intra-tumor heterogeneity and predicting TME interactions. A prior study employing scRNA-seq distinguished between cancerous and non-cancerous cells in samples obtained from patients with breast cancer, revealing both common features within tumors and heterogeneity regarding breast cancer subtypes and key cancer-related pathways [8]. However, these studies have primarily focused on primary breast tumors or other metastatic sites, while the immune landscape of HR + breast cancer with ovarian metastasis remains largely unexplored at the single-cell level. This gap in knowledge is particularly significant given the distinct hormonal environment of the ovary and its potential impact on immune cell function and tumor progression.

The present study was motivated by our clinical observation that patients with HR + breast cancer and ovarian metastasis exhibit distinct clinical trajectories compared to those with other metastatic patterns, yet the underlying cellular and molecular mechanisms remain elusive. Our research team has extensive experience in the surgical management and translational research of breast and gynecological malignancies, which has consistently highlighted the need for a deeper understanding of site-specific metastatic biology. Leveraging this clinical expertise, we employed scRNA-seq to systematically characterize the immune landscape of HR + breast cancer with ovarian metastasis. The primary objective of this investigation was to unravel the cellular dynamics and molecular drivers within this complex microenvironment, with the ultimate goal of identifying potential therapeutic targets to improve outcomes for this underserved patient population.

## Materials and methods

### Tumor sample handling and dissociation to a single-cell suspension for scRNA-Seq

Breast cancer patients with ovarian metastasis who underwent surgery at Cancer Hospital of Shantou University Medical College (Shantou, China) were collected and two fresh surgical specimens (one from the primary tumor and one from paracancerous tissue) were resected by an experienced physician. Fresh samples were washed twice with precooled 0.04% bovine serum albumin (MACS) in RPMI 1640 medium. Each sample for scRNA‐seq was cut into pieces (size≈1 mm^3^) and enzymatically digested with 10 mL of digestion medium containing 0.2% collagenase type IV (Gibco), 0.05% hyaluronidase type I‐S (Sigma–Aldrich), and 0.002% DNase I (Applichem). After 45 min of digestion at 37 °C in a shaking water bath, the enzymatic hydrolysate was filtered through a nylon mesh (pore size: 40 µm) and centrifuged at 1500 rpm for 5 min to obtain a single‐cell suspension. Next, erythrocytes were lysed using a red blood cell lysis solution (MACS) for 5 min. The cell suspension was centrifuged at 1500 rpm for 5 min. After removing the supernatant, the cell pellet was washed twice with RPMI 1640 with 0.04% bovine serum albumin and re‐suspended in the sorting buffer (phosphate-buffered saline supplemented with 0.04% fetal bovine serum). Cell concentration and viability were assessed using a Luna cell counter.

### scRNA-Seq data processing

BD Rhapsody system was used to capture the transcriptomic information of the breast sample-derived single cells. Single-cell capture was achieved by random distribution of a single-cell suspension across > 200,000 microwells through a limited dilution approach. Beads with oligonucleotide barcodes were added to saturation so that a bead was paired with a cell in a microwell. The cells were lysed in the microwell to hybridize mRNA molecules to barcoded capture oligos on the beads. Beads were collected into a single tube for reverse transcription and *Exo* I digestion. Upon cDNA synthesis, each cDNA molecule was tagged on the 5′ end (i.e., the 3′ end of a mRNA transcript) with a unique molecular identifier (UMI) [9] and a cell barcode indicating its cell of origin. Whole transcriptome libraries were prepared using the BD Rhapsody single-cell whole-transcriptome amplification workflow, including random priming and extension, random priming and extension amplification polymerase chain reaction, and whole-transcriptome amplification index polymerase chain reaction. The libraries were quantified using a High Sensitivity DNA chip (Agilent) on a Bioanalyzer 2200 and the Qubit High Sensitivity DNA assay (Thermo Fisher Scientific). Sequencing was performed using an Illumina sequencer (Illumina, San Diego, CA, USA) on a 150-bp paired-end run.

We applied fastp [10] with default parameters filtering the adaptor sequence and removed the low-quality reads to achieve the clean data. UMI-tools [9] was applied for single-cell transcriptome analysis to identify the cell barcode whitelist. The UMI-based clean data were mapped to the human genome (GRCh38) utilizing STAR [11] mapping with customized parameters from the UMI-tools standard pipeline to obtain the UMI counts of each sample. Low-quality cells, namely cells that had fewer than 200 genes or more than 8000 genes expressed, or 20% of UMIs linked to mitochondrial genes, were filtered out.

### Dimensional reduction and unsupervised clustering

R software (Version 3.6.1) and the Seurat package (Version 3.2.0) were used to analyze the output filtered gene expression matrices [12]. In brief, we normalized the cell counts across all cells obtained from the two samples, followed by scaling manipulation and converting to log scale with the function “NomalizeData” by default. Thereafter, the normalized values were used to select highly variable genes (HVGs) with the “FindVariableFeatures” function. Furthermore, the expression profiles of HVGs were converted to z-scores using the function “ScaleData”. Principal components were estimated based on the selected HVGs with the function “RunPCA”, and the first 19 principal components were selected for downstream analysis. The two functions “FindNeighbors” and “FindClusters” were implemented to identify clusters of expression-similar cells with empirically set resolutions, and “resolution = 0.6” was used in the downstream analyses. For visualization, the dimensionality of the dataset was further reduced using Uniform Manifold Approximation and Projection (UMAP) (https://arxiv.org/abs/1802.03426) with the function “RunUMAP”.

### Differentially expressed gene (DEG) identification and functional enrichment

The significantly overexpressed marker genes for clusters were identified using the function “FindAllMarkers” of Seurat. Genes with adjusted *p* value < 0.05 based on the Wilcoxon rank-sum test were defined as cluster-specific signature genes. To identify the potential biological functions of cell clusters, we performed enrichment analysis with signature genes for cell clusters. Kyoto Encyclopedia of Genes and Genomes (KEGG) pathway enrichment analysis and Gene Ontology (GO) term enrichment analysis were performed using R package “clusterProfiler” (Version 3.14.3) with functions “enrichKEGG” and “enrichGO”.

### Monocle trajectory inference analysis

Single-cell pseudotime trajectory analysis was performed using Monocle (Version 2.14.0) [13]. A CellDataSet object was created using the newCellDataSet function with the following parameters: lowerDetectionLimit = 0.1, expressionFamily = negbinomial.size(). Size factors and dispersion parameters were estimated using estimateSizeFactors and estimateDispersions, respectively. Genes expressed in at least five cells (num_cells_expressed ≥ 5) were retained for downstream analysis. Gene detection was performed using detectGenes with min_expr = 0.1. To identify genes associated with cellular heterogeneity, differential expression testing was conducted using differentialGeneTest with the model formula ~ Subtype. Genes were ranked by *p *value, and those with *p* < 0.01 were selected as ordering genes. These genes were specified using setOrderingFilter. Dimensionality reduction was performed using the reduceDimension function with reduction_method = “DDRTree” and max_components = 2. Finally, cells were ordered along the inferred trajectory using the orderCells function to construct the pseudotime trajectory.

### Copy number variation (CNV) analysis

Identification and analysis of malignant cells with CNV estimation for individual cells were conducted using inferCNV (Version 1.2.1) (https://github.com/broadinstitute/infercnv) with a 100-gene sliding window. Genes with an average read count < 0.1 among reference cells were filtered when running inferCNV. T cells were used to define the reference; epithelial cells were used for observations.

### Immunohistochemistry (IHC) staining

Tissue Sects. (4 µm thick) were deparaffinized in xylene and rehydrated through a graded ethanol series. Antigen retrieval was performed by boiling in citrate buffer (pH 6.0) for 15 min or by enzymatic digestion with trypsin for 10 min at 37 °C, depending on the antibody requirements. Endogenous peroxidase activity was blocked with 3% hydrogen peroxide for 10 min, followed by blocking with 5% normal goat serum for 30 min at room temperature. Sections were then incubated overnight at 4 °C with primary antibodies against GPR183 (1:200, 12,377–1-AP, proteintech), BHLHE41 (1:200, 12,688–1-AP, proteintech), CD83 (1:200, 27,873–1-AP, proteintech), SLC25A37 (1:200, 26,469–1-AP, proteintech), and SELL (1:1000, 26,477–1-AP, proteintech). After washing with PBS, sections were incubated with horseradish peroxidase (HRP)-conjugated secondary antibody (1:2000, ab97051, abcam) for 30 min at room temperature. Immunoreactivity was visualized using 3,3'-diaminobenzidine (DAB) chromogen, and sections were counterstained with hematoxylin. Negative controls were prepared by omitting the primary antibody. Images were captured using a light microscope (Olympus BX53) at 200 × or 400 × magnification.

## Results

### Overall characteristics of cell cluster composition in breast cancer with ovarian metastasis and normal tissues

**Fig. 1:**
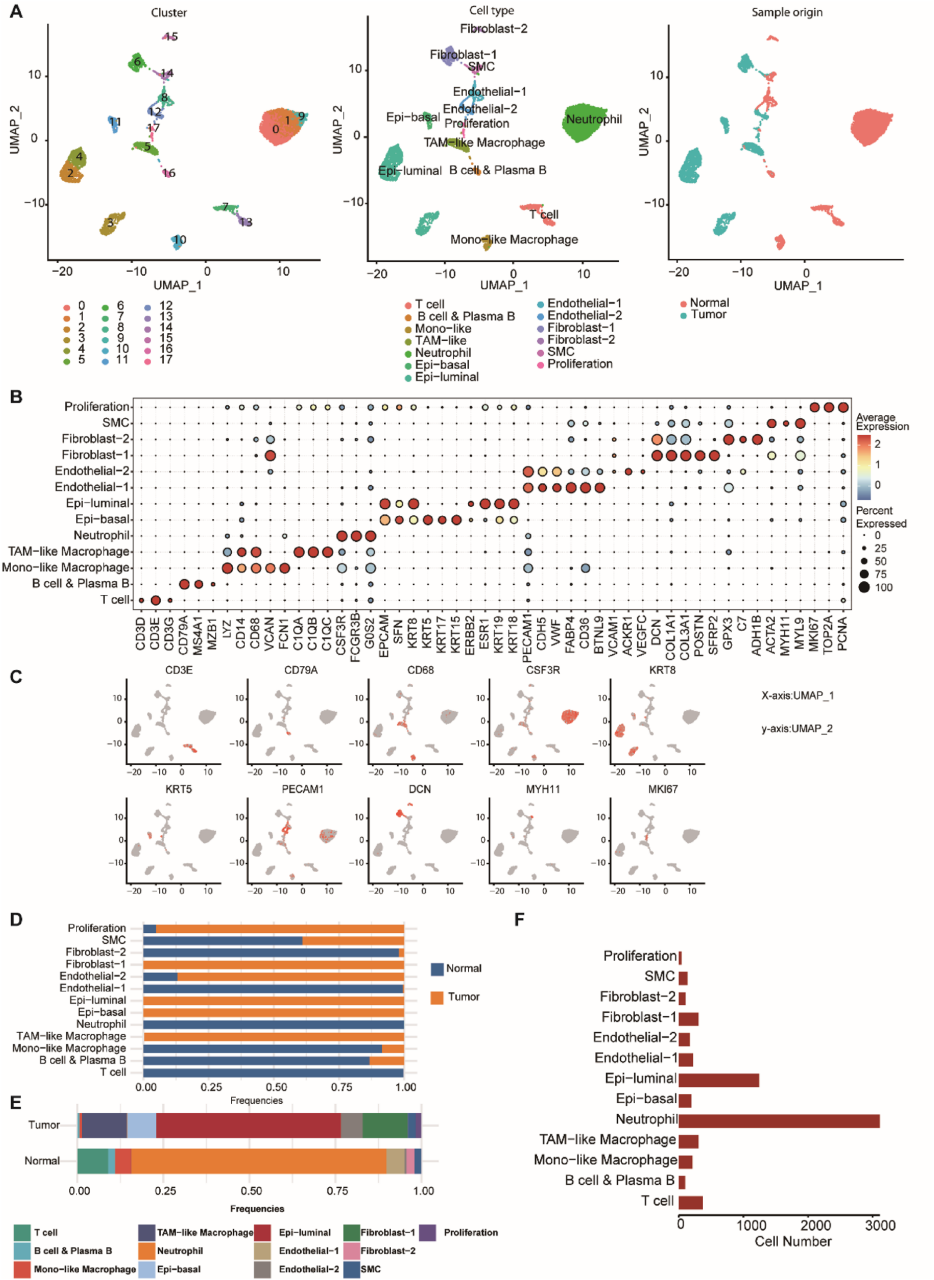
Overview of 6514 single cells obtained from cancer and paracancerous tissue of one breast cancer patient with ovarian metastasis. **A** UMAP plots of the 6514 cells colored according to the 18 clusters and nine major cell types in two samples from one breast cancer patient with ovarian metastases. **B** Expression of classic marker genes used to define the major cell types. **C** Feature plot of representative marker genes for each cell type. **D** Composition ratio of each cell type in non-malignant and tumor samples. **E** Comparison of the percentage of each cell type between non-malignant and tumor samples. **F** Cell numbers of the nine major cell types

A total of 6514 cells from breast cancer tissue (4475 cells) and normal breast tissue adjacent to the cancer (2791 cells) were characterized using scRNA-seq. As shown in Fig. 1A, cells could be grouped into 18 cell clusters (clusters 0–17), within which nine cell types were identified based on the marker genes. In addition, a total of 1238 cells in clusters 2–4 were comprised of breast epithelial cells, accounting for 19.01% of the total number of cells. Cluster 11 was composed of basal cells (185, 2.84%), and Clusters 8 and 12 were composed of endothelial cells (relative proportion: 6.86%). Myeloid cells comprised clusters 0, 1, 9 (neutrophils, 2894, 44.43%), and 5, 10 (macrophages, 616, 9.46%), respectively. Clusters 6 and 15 were composed of fibroblasts (461, 7.08%), and clusters 16 and 7, 13 represented B cells (40, 1.5%) and T cells (511, 7.84%), respectively. Among normal tissues, the predominant cell populations were neutrophils (66.90%), macrophages (10.76%), and T cells (7.61%). Compared with normal tissues, luminal epithelial cells (53.83%), fibroblasts (13.12%), and macrophages (12.39%) were more abundant in tumor tissues. Moreover, the proportions of neutrophils (3.92%), T cells (0.90%), and B cells (0.99%) were lower than those detected in normal samples. The feature plot of the representative marker genes for each cell type cluster is shown in Fig. 1B and C. The frequency of each cell type in tumor and normal samples, the relative proportions of different cell types in carcinoma and paracancerous samples, and the number of cells in each of the nine major cell types are shown in Fig. 1D, E, and F, respectively.

### Epithelial cell heterogeneity in breast cancer and paracancerous tissues

**Fig. 2:**
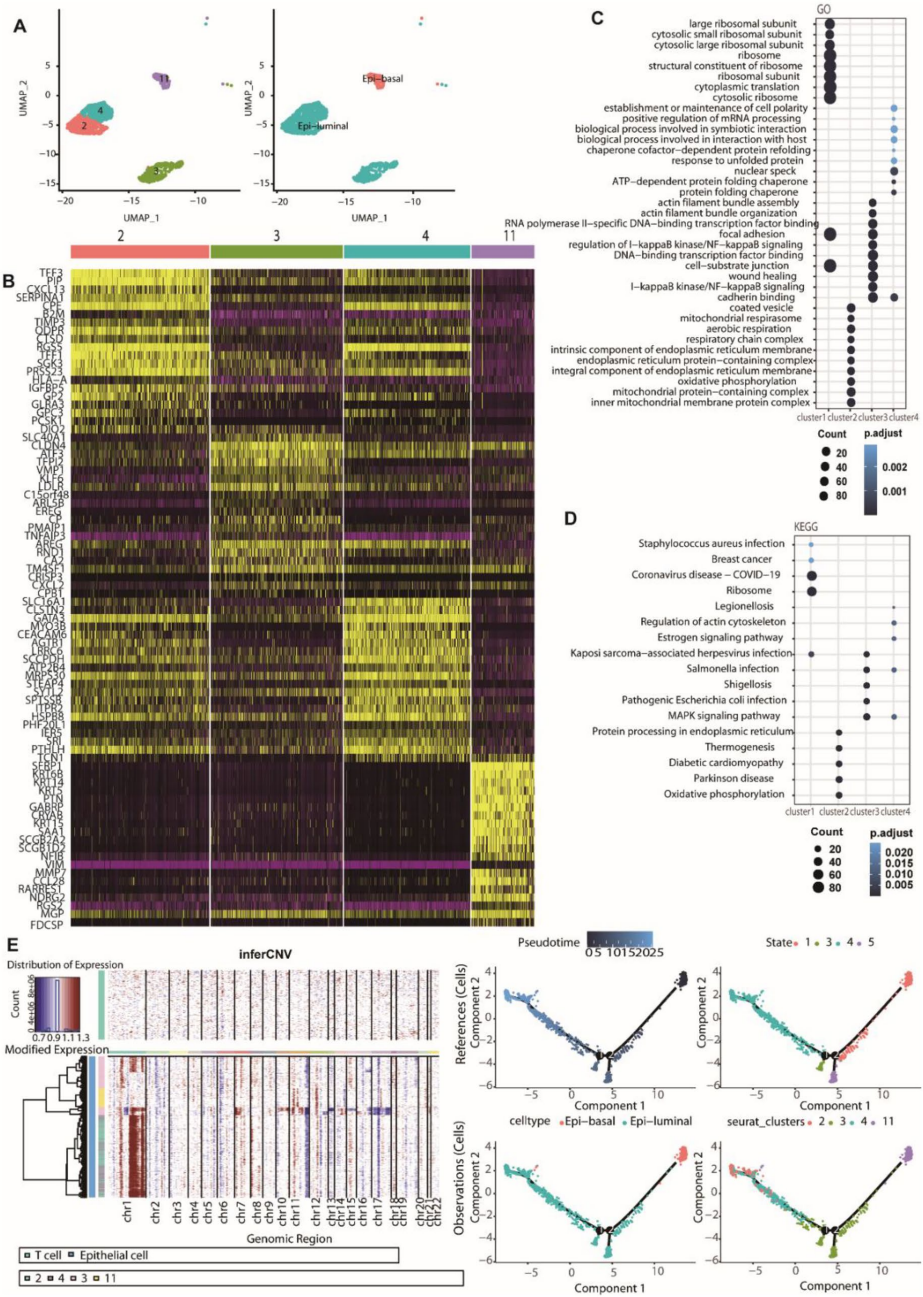
Epithelial cell subclusters in breast cancer. **A** UMAP embedding of epithelial cells reveals four clusters and two main subpopulations, luminal epithelial cells and basal epithelial cells, arranged from left to right. **B** Heatmap showing DEGs with a Log2 (fold change) of > 0.5 between different clusters. **C** The top 10 enriched GO biological process terms of each epithelial cell subcluster. **D** The top 5 enriched KEGG pathways of the epithelial cell clusters. **E** InferCNV heatmap for epithelial cells showing copy number variations in the chromosomes across four clusters. T cells were used as references (top). Red and blue represent amplification and deletion, respectively. **F** Pseudotime analysis of epithelial cell differentiation trajectory in the breast cancer sample. Color key, transitioning from dark to bright, represents the progression of cancer from the early stage to the late stage

Breast cancer originates from genetic changes in breast epithelial cells, resulting in the disruption of tissue balance [14]. Understanding the properties and functions of these cells will help understand the origin and progression of cancer. An extensive analysis across non-malignant and tumor samples identified 1445 epithelial cells, which were classified into four distinct subclusters (Fig. 2A and B). Breast epithelial cells were classified by two cytokeratins: keratin 15/17 (KRT15/17) and KRT5 were used for basal markers, and KRT8/18 and KRT19 were used for luminal markers. In addition, stratifin (SFN) was another marker gene that is usually highly expressed in the luminal subtype [15].

Subsequently, we identified the top 10 enriched GO terms pathways for upregulated genes within different epithelial cell clusters, along with the top five KEGG pathways (Fig. 2C and D). Cluster 3 was enriched in two important tumor immunity-related biological process GO terms: “I-kappaB kinase/NF-kappaB signaling” and “regulation of I − kappaB kinase/NF − kappaB signaling” (Fig. 2C). As shown in Fig. 2D, the “MAPK signaling pathway” was significantly enriched in both clusters 3 and 4 epithelial cell subsets. Reports have shown that the MAPK signaling pathway plays important roles in cell proliferation, survival and metastasis, even in the development of breast cancer [16].

Studies have shown that studying CNVs in breast cancer and adjacent normal tissues may provide clues to understanding the development of breast cancer [17]. Furthermore, to reveal the heterogeneity within tumor epithelial cells, we conducted CNV detection to deduce extensive alterations in copy numbers across various chromosomes within individual cells (Fig. 2E). In our data, there was a higher frequency of gain on chromosome 1, while loss was more commonly detected on chromosomes 11 and 17. Among different subpopulations of epithelial cells, the most CNVs occurred in clusters 2 and 4, followed by cluster 3, whereas the fewest CNVs occurred in cluster 11. Pseudotime analysis was performed to generate pseudotime trajectories of epithelial cell development, primarily composed of two key cell types: epithelial basal cells and luminal cells (Fig. 2F). Tracing the pseudotime trajectory showed that basal cells were present at the initial stage, corresponding to the cluster 11 subset. The luminal epithelial cells were mainly located in the middle and late stages of the trajectory. The intermediate stage primarily consisted of the cluster 3 subset, while the late stage of the trajectory was a composite of the cluster 2 and cluster 4 subsets (Fig. 2F). This corresponds with the observation in Fig. 2E, where CNVs were primarily concentrated within the cluster 2 and cluster 4 subsets. Genetic alterations have the potential to initiate the transformation of breast tissue cells into cancerous forms. Reconstructing the developmental pathways of breast cells can enhance our comprehension of cancer initiation and progression.

### Macrophage heterogeneity in breast cancer and paracancerous tissues

**Fig. 3:**
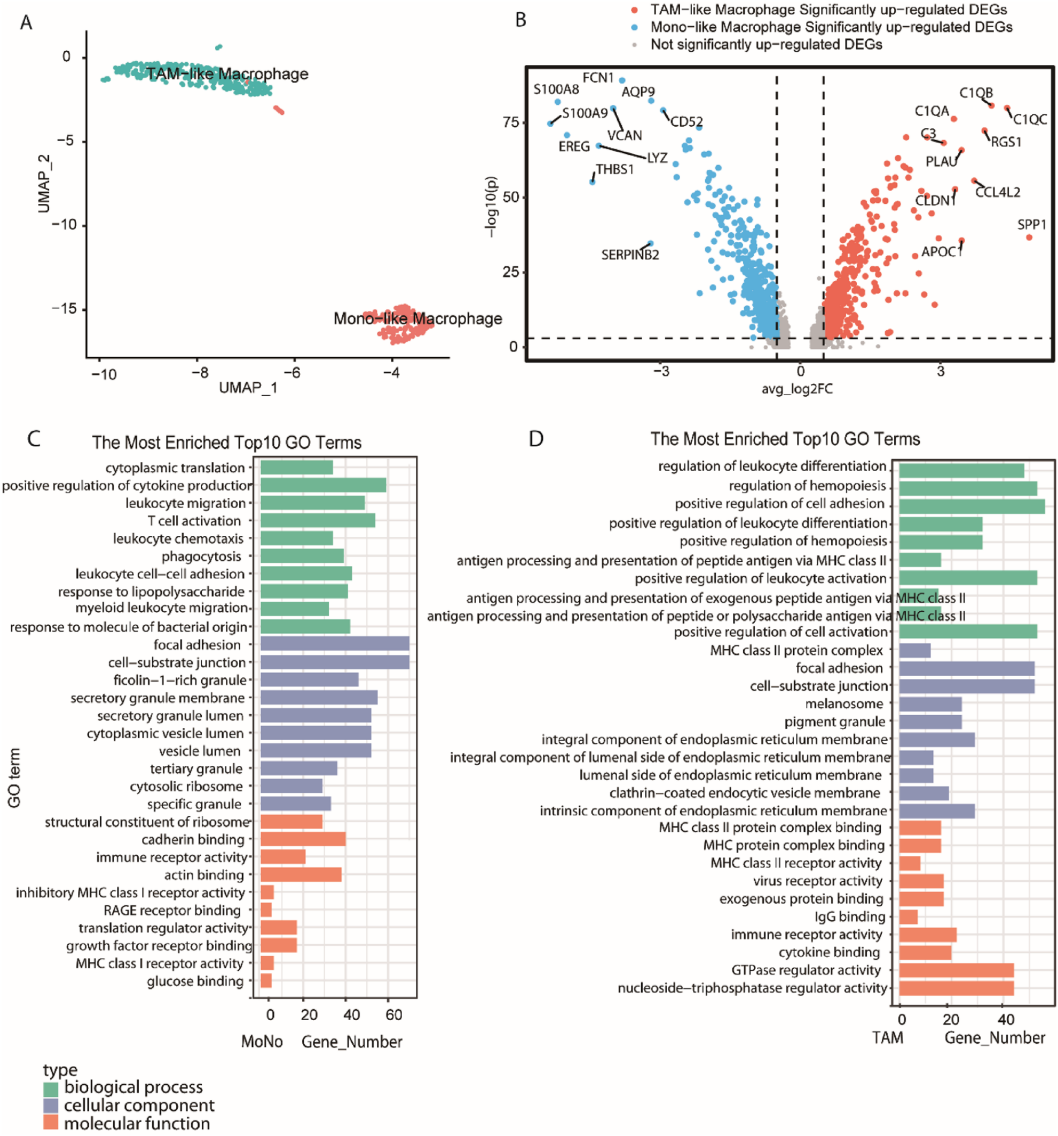
Macrophage heterogeneity and functional enrichment in breast cancer. **A** UMAP visualization of the clustering of 516 macrophage cells from non-malignant and tumor samples. **B** Volcano plot of significantly upregulated DEGs between TAM-like and MONO-like macrophage cell subpopulations (blue and red indicate MONO-like and TAM-like macrophages, respectively). **C** The Most Enriched Top 10 GO Terms for Mono-like macrophages. Bar plots show the number of enriched genes categorized by biological process (green), cellular component (blue), and molecular function (orange). **D** The Most Enriched Top 10 GO Terms for TAM-like macrophages. Terms related to leukocyte differentiation and hemopoiesis are prominent. (Note: detailed marker gene expression and KEGG pathway enrichment analyses are provided in Supplementary Figs. 1 and 2.)

Macrophages are vital drivers of tumor-promoting inflammation [18]. From the cancer and normal samples, we detected a total of 739 macrophage cells. An integrated analysis identified two distinct subclusters: monocytes, comprising 212 cells, defined as 'Mono-like', and TAMs, consisting of 304 cells, defined as 'TAM-like' (Fig. 3A). Transcriptional profiling revealed significant divergence between these two subsets. The volcano plot (Fig. 3B) highlights distinct differentially expressed genes (DEGs), with S100A8 and S100A9 characterizing the Mono-like subset, while APOC1 and C1QA marked the TAM-like subset. The expression patterns of the top 50 marker genes further confirmed these identities (Supplementary Fig. 1).

To understand the functional implications of this heterogeneity, we performed Gene Ontology (GO) and KEGG enrichment analyses. In the GO analysis, Mono-like macrophages were significantly enriched in pathways related to positive regulation of cytokine production and T cell activation (Fig. 3C), suggesting a potential role in immune activation. In contrast, TAM-like macrophages were primarily associated with the regulation of leukocyte differentiation and hemopoiesis (Fig. 3D), consistent with an immunomodulatory phenotype. Consistent with these findings, KEGG pathway analysis (Supplementary Fig. 2) revealed that Mono-like macrophages were enriched in Natural killer cell mediated cytotoxicity and Osteoclast differentiation, whereas TAM-like macrophages showed significant enrichment in Antigen processing and presentation and Phagosome pathways. These distinct functional profiles underscore the plasticity of macrophages in breast cancer.

### Dynamic gene expression profiling of macrophages in breast cancer

**Fig. 4:**
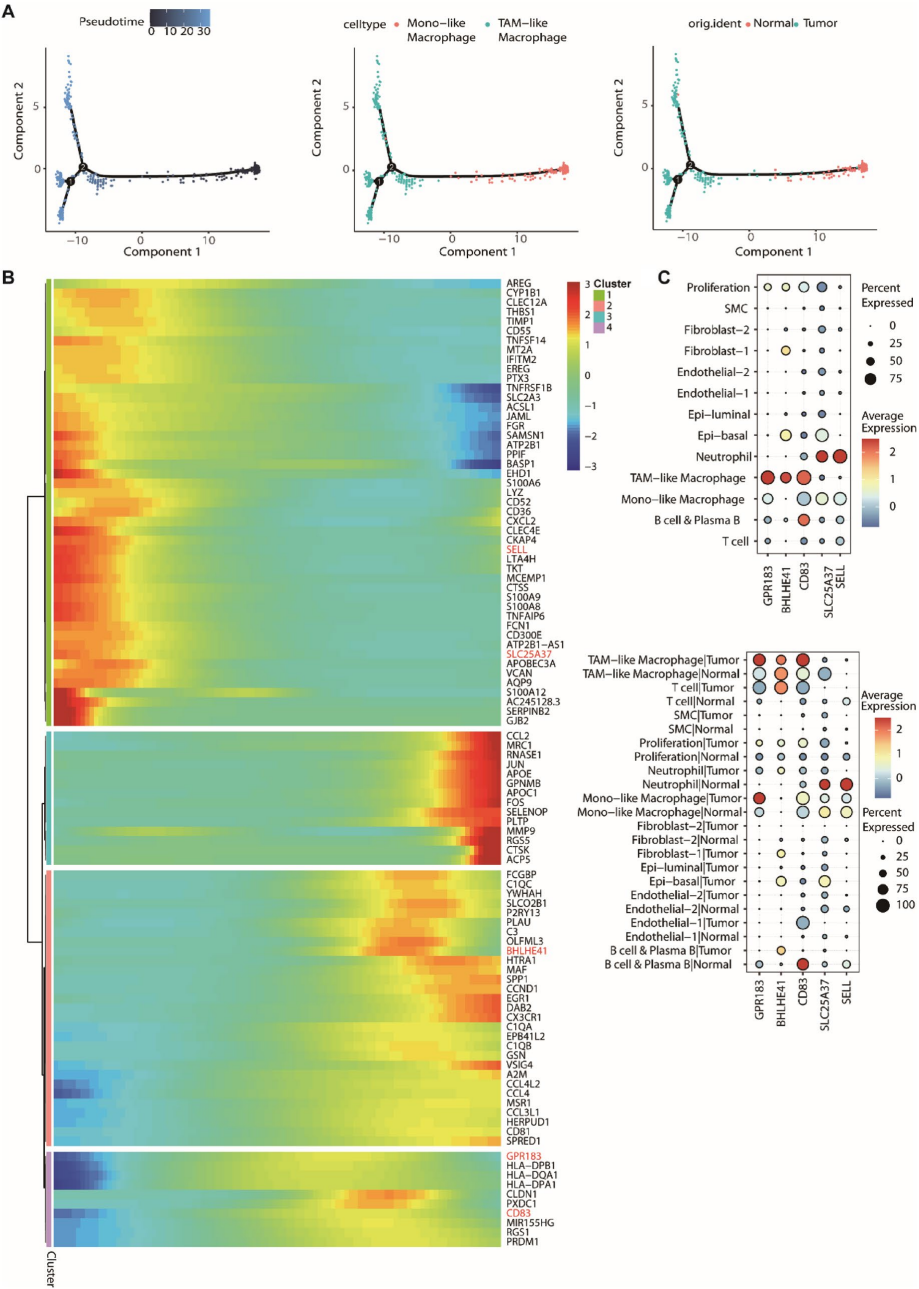
Pseudotime trajectory analysis and dynamic gene expression profiling of macrophages in breast cancer. **A** Pseudotime trajectory of macrophage differentiation. Left: cells ordered along the pseudotime axis. Middle: trajectory colored by cell type (Mono-like vs. TAM-like), showing a transition from Mono-like to TAM-like states. Right: trajectory colored by sample origin (Normal vs. Tumor), indicating that normal cells primarily occupy the early stage while tumor cells dominate the late stage. **B** Heatmap illustrating the top 100 dynamically expressed genes along the pseudotime trajectory. Genes were grouped into four distinct clusters (Clusters 1–4) based on their expression trends. The color key from blue to red indicates relative expression levels from low to high. **C** Dot plots visualizing the expression of five prognosis-related genes (GPR183, CD83, BHLHE41, SLC25A37, and SELL) identified through a multi-step screening. Candidates were selected from the top 100 dynamic genes, filtered for differential expression between Mono-like and TAM-like macrophages (avg _log2FC > 0, *p* < 0.05), and validated by survival analysis. Top panel: expression profiles across distinct cell clusters, demonstrating cell-type specificity. Bottom panel: expression profiles stratified by both cell type and tissue origin (Normal vs. Tumor), revealing microenvironment-dependent regulation. Dot size represents the percentage of cells expressing the gene, and color intensity represents the average expression level

Pseudotime analysis was conducted to establish pseudotime paths for breast cancer macrophages. Based on the pseudotime trajectory, MONO-like macrophage cells were mainly found in the early stages of the trajectory, while TAM-like macrophages were predominantly situated in the later stages (Fig. 4A). Similarly, early-stage cells within the trajectory primarily stemmed from normal samples, whereas cells from tumor samples were primarily localized in the later stages, suggesting a reprogramming process from normal/monocytic states to tumor-associated states (Fig. 4A). To uncover the molecular drivers of this transition, we analyzed dynamic gene expression patterns along the trajectory (Fig. 4B). We identified the top 100 genes with the most significant expression changes and clustered them into four modules. The heatmap revealed distinct trends: genes associated with inflammation and monocyte identity (e.g., *SELL, CXCL2, S100A8*) were highly expressed in the early stages but downregulated as cells progressed. In contrast, genes enriched in the late stages included *GPR183*, *BHLHE41*, and *CD83*, which peaked in TAM-like macrophages. This shift highlights the transcriptional reprogramming macrophages undergo to adapt to the tumor microenvironment.

To identify robust biomarkers, we applied a stringent multi-step filtering strategy to the top 100 dynamic genes. First, we retained genes showing significant differential expression between Mono-like and TAM-like macrophages (*p* < 0.05, avg_log2FC > 0). From this refined subset, we further used GEPIA2 (Gene Expression Profiling Interactive Analysis 2) survival analysis to screen for genes that significantly impact survival specifically in breast cancer samples. We finally selected five candidates (GPR183, CD83, BHLHE41, SLC25A37, and SELL) that exhibited robust dynamic changes, significant cell-type specificity, and strong association with patient survival. Their expression patterns are depicted in Fig. 4C in two dimensions. The upper panel confirmed the cell-type specificity of these genes, showing they were predominantly expressed in macrophage subsets rather than lymphocytes. The lower panel further stratified the data by tissue origin (Normal vs. Tumor), revealing that the expression of these markers is not only cell-type dependent but also significantly modulated by the tumor microenvironment. Specifically, GPR183, BHLHE41, and CD83 exhibited high expression in TAM-like macrophages and low expression in MONO-like macrophages. In contrast, SLC25A37 and SELL were highly expressed in MONO-like macrophages and had low expression in TAM-like macrophages.

**Fig. 5:**
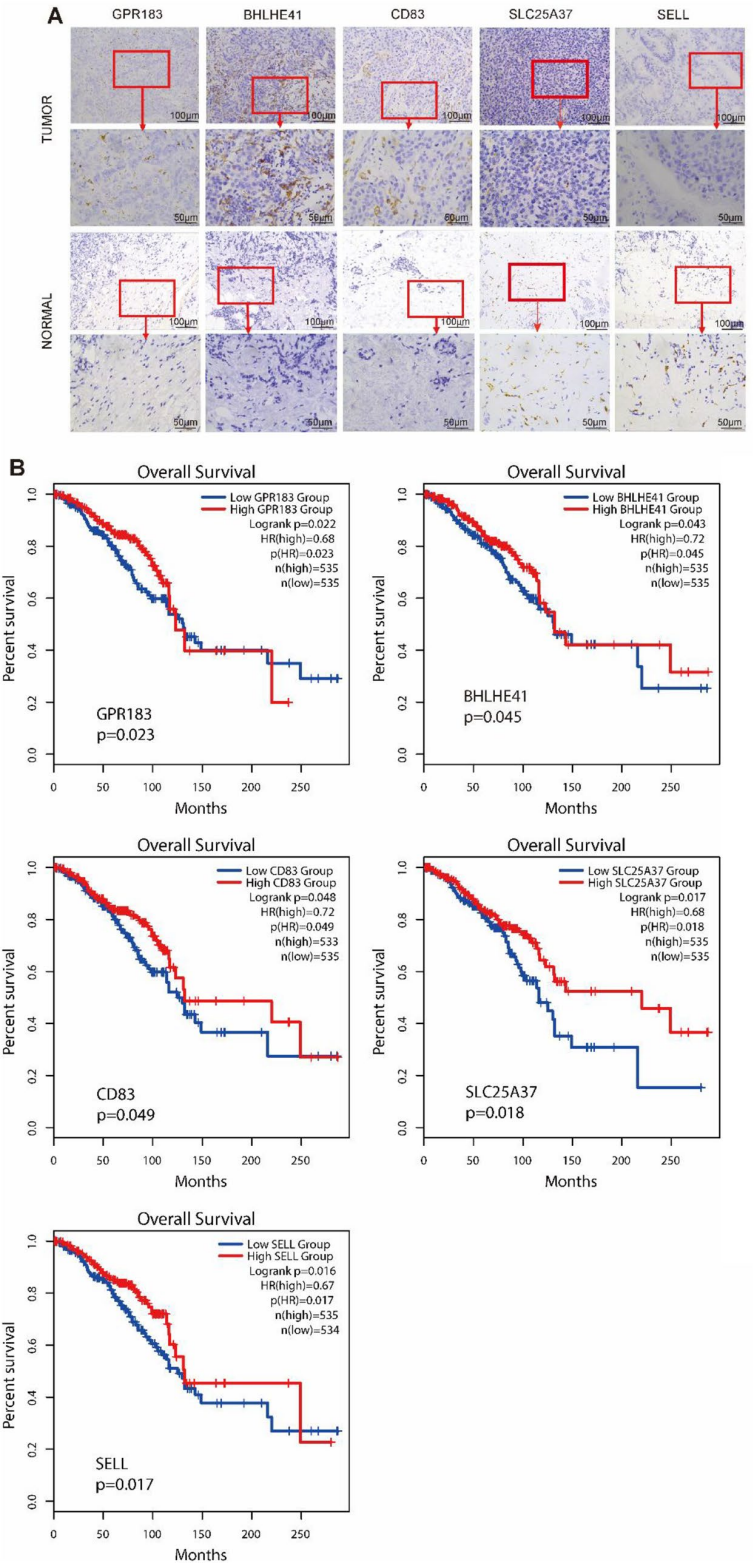
Correlation of overall survival with GPR183, BHLHE41, CD83, SLC25A37, and SELL expression in breast invasive carcinoma (BRCA). **A** Immunohistochemical detection of GPR183, BHLHE41, CD83, SLC25A37, and SELL expression in tumor and adjacent normal tissues of breast cancer patients with ovarian metastases. **B** Associations of GPR183, BHLHE41, CD83, SLC25A37, and SELL with survival and prognosis were analyzed using GEPIA2. Blue and red represent low and high gene expression groups, respectively. All Kaplan–Meier curves exhibited a positive correlation

Immunohistochemical staining results of breast cancer tissues and normal adjacent tissues obtained from 10 breast cancer patients with ovarian metastases showed that GPR183, BHLHE41, and CD83 were highly expressed in cancer tissues and lowly expressed in normal adjacent tissues, while SLC25A37 and SELL were lowly expressed in cancer tissues and highly expressed in normal adjacent tissues (Fig. 5A). IHC staining showed increased protein expression in tumor tissues, consistent with macrophage infiltration patterns, though we cannot exclude a contribution from other stromal cell types without additional marker analysis. We also analyzed the relationship of GPR183, CD83, BHLHE41, SLC25A37, and SELL expression in breast invasive carcinoma with patient survival and prognosis (Fig. 5B). The analysis showed that high GPR183, CD83, BHLHE41, SLC25A37, and SELL expression was associated with a higher overall survival rate compared with low expression. In the mid-term, the survival rates of the two groups converged.

## Discussion

Macrophages, particularly TAMs, are key players in tumor-associated inflammation and progression. In the TME, TAMs are enriched in immune suppression and metabolic reprogramming pathways. They promote immune checkpoint molecules (e.g., PD-L1) and secrete cytokines (e.g., IL-10 and TGF-β), creating an immunosuppressive environment that inhibits cytotoxic T-cell activation [18, 19]. Targeting these pathways with agents, such as immune checkpoint inhibitors, has shown promise in reversing TAM-mediated immune evasion [6, 20, 21]. TAMs also exhibit enhanced glycolysis and fatty acid oxidation, supporting tumor growth and angiogenesis [7, 22]. These macrophages can be divided into M1 TAMs (pro-inflammatory and anti-tumor) and M2 TAMs (immunosuppressive, pro-tumor, and pro-angiogenic). The metabolic profiles of M1 and M2 TAMs differ significantly, with M1 TAMs predominantly relying on glycolysis and M2 TAMs favoring fatty acid oxidation and oxidative phosphorylation [23]. Inhibiting metabolic pathways, such as fatty acid oxidation or arginine metabolism, holds therapeutic potential in disrupting TAM functions [24, 25]. Targeting TAM-enriched pathways can transform the TME into an anti-tumor environment [26].

In HR + metastatic breast cancer, TAMs promote tumor growth and metastasis, with key genes (e.g., GPR183, BHLHE41, and CD83) associated with poor survival [13, 26, 27]. In contrast, macrophages derived from monocytes (MONO-like macrophages) exhibit functions that are generally associated with tissue repair and inflammation [28]. Unlike TAMs, MONO-like macrophages participate in early-stage immune responses and are less likely to directly support tumor growth and metastasis [29]. They tend to express genes such as SLC25A37 and SELL, which are linked to normal immunological functions rather than tumor progression [22]. The functional dichotomy between these two macrophage types presents potential therapeutic targets. Strategies that shift the macrophage population balance from TAMs to a more M1-like phenotype could suppress tumor progression and metastasis [24]. Therefore, understanding the specific roles and molecular pathways associated with these macrophage types is crucial for developing targeted therapies that could disrupt the pro-tumor functions of TAMs, while enhancing the anti-tumor capabilities of the immune system [30]. Our study aimed to elucidate the complex interplay between different cell types within the TME of HR + metastatic breast cancer, with a particular focus on the roles of macrophages. The identification of diverse macrophage populations, including TAMs and MONO-like macrophages, has provided a deeper understanding of their distinct roles in promoting tumor growth and metastasis.

Breast cancer arises from the breast epithelium, which is composed of two known cell types, an inner layer of secretory luminal cells and an outer layer of basal/myoepithelial cells [31]. In our study, we identified four distinct subclusters between non-malignant and tumor samples, involving basal markers KRT15/17 and KRT5, and luminal-specific KRT8/18 and KRT19. KRT15 is correlated with human epidermal growth factor receptor 2 (HER2) positivity, pathological grade, and N stage in breast cancer [32]. Reduced expression of KRT17 predicts poor prognosis in HER2 (high) breast cancer [33]. Expression of KRT17 and/or KRT5/6 in tumor cells is associated with a poor clinical outcome in breast carcinoma [34].

Macrophages are diverse immune cells that play an essential role in the breast during homeostasis and cancer [35]. We also conducted an integrated analysis of this compartment in both non-malignant and tumor samples, and identified two distinct MONO-like and TAM-like subclusters. We also identified many macrophage markers. The TOP 50 DEGs between MONO-like macrophages and TAM-like macrophages were analyzed, and the GO terms were also discussed.

Furthermore, pseudotime analysis showed that the MONO-like macrophages are mainly found in the early stages of the trajectory, while TAM-like macrophages are predominantly situated in the later stages. Gene expression analysis revealed that three genes (GPR183, BHLHE41, and CD83) are highly expressed in TAM-like macrophages, and two genes (SLC25A37 and SELL) are highly expressed in MONO-like macrophages. GPR183 is involved in inflammatory autoimmune diseases [24]. GPR183 and CD83 are markers expressed in TAMs, contributing to their pro-tumorigenic roles. GPR183, also known as EBI2, is a G protein-coupled receptor involved in inflammatory responses and immune cell positioning. Its overexpression in TAMs enhances inflammatory signaling pathways, which support tumor cell proliferation and immune evasion [18, 24]. Studies have shown that GPR183 expression is correlated with poor survival outcomes in breast cancer, particularly in HR + subtypes [13, 27]. GPR183 plays a significant role in the prognosis of breast cancer, particularly in HR + subtypes. High expression of GPR183 in TAMs has been correlated with poor survival outcomes in patients with breast cancer, highlighting its potential as a prognostic biomarker [24, 27]. Its prognostic significance stems from its involvement in key tumor-promoting processes, including immune suppression, angiogenesis, and tumor cell motility. The pro-tumorigenic effects of GPR183 are mediated through its regulation of inflammatory and chemotactic signaling pathways. GPR183 interacts with oxysterols in the TME, facilitating the recruitment and activation of immune cells, particularly TAMs, which support tumor growth and metastasis [18]. Additionally, GPR183 enhances the expression of pro-inflammatory cytokines and chemokines, such as CCL4, that further modulate the immune microenvironment to favor cancer progression [36]. Involvement of the receptor in metabolic reprogramming, particularly fatty acid oxidation and glycolysis in TAMs, provides additional support for its role in fostering a pro-metastatic niche [7]. GPR183 expression may influence cancer therapy responses by modulating the tumor immune landscape. High levels of GPR183 in TAMs are associated with increased immune suppression, which could reduce the efficacy of immunotherapies such as checkpoint inhibitors. Conversely, targeting GPR183 could potentially reprogram TAMs into an anti-tumor phenotype, thus improving treatment responses [26, 30]. Furthermore, the role of GPR183 in promoting metastatic niches suggests that its inhibition could reduce the likelihood of long-term metastasis, thereby improving survival rates. In conclusion, GPR183 may serve as a critical marker for breast cancer prognosis and represent a promising therapeutic target. Nonetheless, further research is warranted to investigate its role in therapy resistance and its potential as a predictive biomarker for patient outcomes.

Similarly, CD83 is an immunoglobulin superfamily protein primarily associated with dendritic cell maturation; however, it is also expressed in TAMs. High levels of CD83 in TAMs are linked to the modulation of immune responses, promoting an immunosuppressive environment that facilitates tumor progression [26, 29]. Moreover, CD83 expression is indicative of enhanced TAM recruitment and activity, which drives angiogenesis and metastasis [30].These findings emphasize the importance of GPR183 and CD83 as key regulators of TAM-mediated tumor-promoting activities. Their expression profiles provide potential prognostic biomarkers and therapeutic targets for disrupting TAM functions in the TME [19].

Basic helix-loop-helix family member e41 (BHLHE41) facilitates MCF-7 cell invasion mainly via activation of the MAPK/JNK signaling pathway [26]. Dense CD83 + dendritic cell infiltration was observed in luminal tumors of breast carcinoma [13]. Overexpression of SLC25A37, a member of the solute carrier family, causes cancer-related fatigue in patients with non-metastatic prostate cancer during external beam radiation therapy [22]. SELL binding induces activation of leukocytes, which can be further modulated by selectin-mediated interactions with platelets and endothelial cells [37]. The expression of these proteins was confirmed using immunohistochemistry with breast cancer tissues and normal adjacent tissues. The prognostic value of these proteins was also evaluated using the GEPIA2 website.

There were discrepancies and limitations in our study. GPR183, CD83, and BHLHE41 were highly expressed in TAM-like macrophages and abundant in tumor tissues. However, our survival analysis unexpectedly revealed that high expression of these genes was associated with improved patient survival, which appears contradictory to their canonical pro-tumor functions. We believe this apparent discrepancy can be explained by the complex tumor microenvironment in breast cancer. The results that high expression of GPR183, CD83, and BHLHE41 predicted the improved prognosis of the patients were based on the examination of total transcript abundance of these genes in breast cancer tissues. As the microenvironment in breast cancer tissues had various kinds of cell types, including tumor cells, macrophages, cancer-associated fibroblasts and other types of immune cells [38], the total expression of these genes could not reflect the precise function of these genes in specific cell types. The expression pattern of the same gene in different cell types may exhibit disparate functions, either tumor-promoting or tumor-suppressing, in breast cancer. For example, Caveolin‑1 (Cav‑1), a key structural component of plasma membrane caveolae, is critically implicated in breast cancer metastasis. Functionally, Cav‑1 acts as a double-edged sword, exerting either tumor‑suppressive or oncogenic effects that are highly dependent on the cellular microenvironment and breast cancer subtype [39]. Thus, whether overexpression of GPR183, CD83, and BHLHE41 in different cell types promotes or suppresses breast cancer development remains to be determined. We will perform experiments to explore the precise role of these genes in the follow-up study.

The findings of this study provide significant insights compared with previous single-cell research on cancer. A study conducted in 2024 identified new exhaustion markers, such as CXCL13 and LAYN, in CD8 + T cells [40]. The investigation focused on the functional states of T cells and their anti-tumor potential within the TME, offering novel targets for immunotherapy. In contrast, the present study delves into the functional differences between TAMs and monocyte-derived macrophages, particularly in HR + breast cancer with ovarian metastasis. Notably, this research highlights the key roles of TAMs in promoting immune suppression and metabolic reprogramming. We also report, for the first time, the specific high expression of GPR183 and CD83 in TAMs, linking these genes to poor patient outcomes. These findings extend the scope of single-cell analysis from the traditional focus on T cell heterogeneity to the functional roles of macrophages, emphasizing the central role of TAMs in tumor progression and metastasis. Moreover, unlike prior studies that focus on the functions of a single cell type, this study uses pseudotime analysis to illustrate the dynamic transition of MONO-like macrophages into TAMs. While pseudo-time analysis predicts continuous transcriptional changes during macrophage polarization, our IHC validation is limited to dichotomous normal-tumor comparisons. Future studies incorporating multi-region sampling across tumor stages or utilizing spatial transcriptomics would provide more comprehensive validation of the inferred trajectory. Furthermore, definitive attribution of IHC signals to specific cell types requires validation beyond single-marker staining. We suggest that future studies employ multiplex immunofluorescence (combining target genes with macrophage markers CD68/CD163) or in situ hybridization to conclusively establish cellular specificity.

In summary, this study advances the understanding of macrophage heterogeneity and function in HR + metastatic breast cancer, highlighting TAM-specific pathways (e.g., immune suppression and metabolic reprogramming) as key drivers of tumor progression. By identifying GPR183 and CD83 as potential therapeutic targets, this research provides a foundation for developing innovative treatments. Future directions include validating these findings in patient-derived xenograft models, exploring TAM-targeting drugs, and designing combination therapies to modulate the TME. Such efforts should aim to translate these insights into precision medicine approaches to improve patient outcomes.

## Supplementary Information

Below is the link to the electronic supplementary material.

Supplementary file1 (DOCX 720 KB)

## Data Availability

The data are available from the corresponding author upon reasonable request.
