# Supplementary material for: Deciphering the immunological landscape of HR + metastatic breast cancer: insights from single-cell transcriptomics
Source: Hum Cell. 2026 May 8;39(5):73. doi: 10.1007/s13577-026-01384-2 (PMC13156106; doi:10.1007/s13577-026-01384-2)
Supplement: Supplementary file 1 — Supplementary file1 (DOCX 720 KB) [file 13577_2026_1384_MOESM1_ESM.docx]

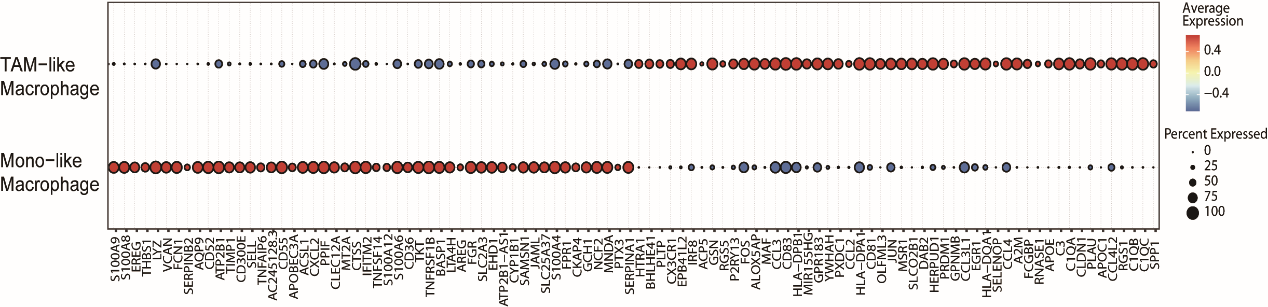


**Supplementary Figure 1. Cell-type specific expression of marker genes in macrophage subsets.** Dot plots showing the expression of the top 50 differential genes in each macrophage subpopulation. Dot size represents the percentage of cells expressing the gene, and color intensity represents the average expression level (Red: high expression, Blue: low expression).


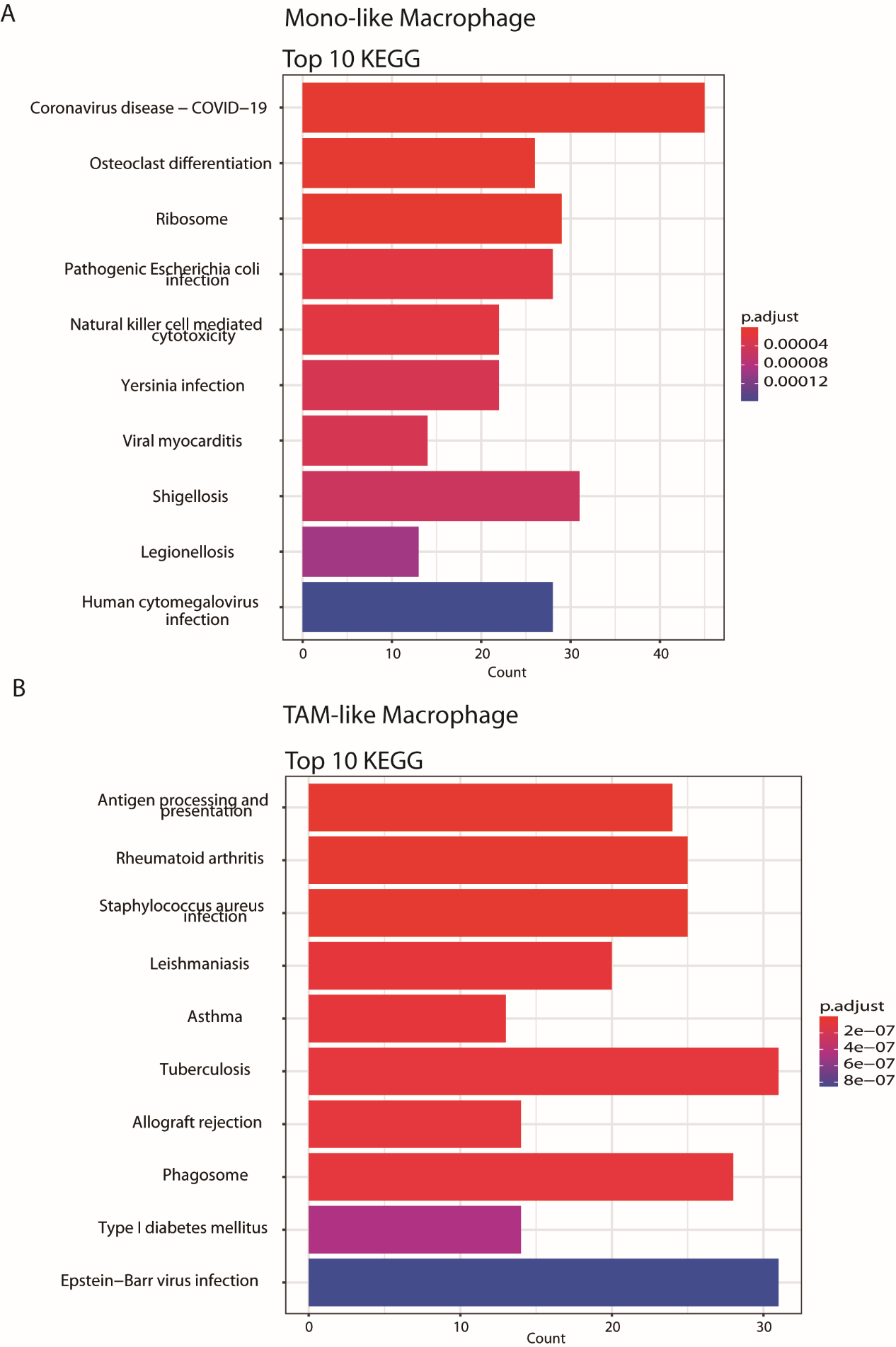


**Supplementary Figure 2. KEGG pathway enrichment analysis of macrophage subsets. (A) Top 10 enriched KEGG pathways for Mono-like macrophages.** Pathways related to immune cytotoxicity and differentiation are highlighted. (B) Top 10 enriched KEGG pathways for TAM-like macrophages.  Pathways related to antigen processing and phagocytosis are prominent. Color intensity represents the adjusted P-value (p.adjust), and bar length indicates the gene count.
